# Empirical Antibiotic Therapy for Gram-Negative Bacilli Ventilator-Associated Pneumonia: Observational Study and Pharmacodynamic Assessment

**DOI:** 10.3390/antibiotics11111664

**Published:** 2022-11-19

**Authors:** Olivier Pajot, Karim Lakhal, Jérome Lambert, Antoine Gros, Cédric Bruel, Thierry Boulain, Denis Garot, Vincent Das, Jean François Timsit, Charles Cerf, Bertrand Souweine, Cendrine Chaffaut, Hervé Mentec, Jean Ralph Zahar, Jean Paul Mira, Vincent Jullien

**Affiliations:** 1 Victor Dupouy Hospital, Intensive Care Unit, F-95100 Argenteuil, France; 2 Service d’Anesthésie-Réanimation, Hôpital Laënnec, Centre Hospitalier Universitaire, F-44093 Nantes, France; 3 Department of Biostatistics and Medical Information, APHP, Saint-Louis Hospital, F-75010 Paris, France; 4 Medical-Surgical Intensive Care Unit, André Mignot Hospital, F-78150 Le Chesnay, France; 5 Medical and Surgical Intensive Care Unit, Paris Saint-Joseph Hospital Network, F-75014 Paris, France; 6 Intensive Care Unit, Orleans Regional Hospital, 14 Avenue de L’Hôpital CS 86709, CEDEX 02, F-45067 Orléans, France; 7 Service de Médecine Intensive Réanimation, Hôpital Bretonneau, CHU Tours, F-37000 Tours, France; 8 Service de Médecine Intensive Réanimation, Centre Hospitalier Intercommunal André Grégoire, F-93100 Montreuil, France; 9 AP-HP, Bichat Hospital, Medical and Infectious Diseases Intensive Care Unit (MI2), F-75018 Paris, France; 10 Intensive Care Unit, Foch Hospital, F-92150 Suresnes, France; 11 CHU Clermont-Ferrand, Service de Réanimation Médicale, F-63000 Clermont-Ferrand, France; 12 AP-HP, Hôpital Avicenne, Prévention du Risque Infectieux, GH Paris Seine Saint-Denis, F-93000 Bobigny, France; 13 Department of Medical Intensive Care, Cochin University Hospital, F-75014 Paris, France; 14 Pharmacology Unit, University Sorbonne Paris Nord, Groupe Hospitalier Paris Seine-Saint-Denis, Assistance Publique-Hôpitaux de Paris, Hôpital Jean Verdier, F-93140 Bondy, France

**Keywords:** VAP, pharmacokinetics, pharmacodynamics, intensive care unit

## Abstract

Background: Strong evidence suggests a correlation between pharmacodynamics (PD) index and antibiotic efficacy while dose adjustment should be considered in critically ill patients due to modified pharmacokinetic (PK) parameters and/or higher minimum inhibitory concentrations (MICs). This study aimed to assess pharmacodynamic (PD) target attainment considering both antibiotics serum concentrations and measured MICs in these patients. Method: A multicentric prospective open-label trial conducted in 11 French ICUs involved patients with Gram-negative bacilli (GNB) ventilator-associated pneumonia (VAP) confirmed by quantitative cultures. Results: We included 117 patients. Causative GNBs were *P. aeruginosa* (40%), *Enterobacter* spp. (23%), *E. coli* (20%), and *Klebsiella* spp. (16%). Hence, 117 (100%) patients received β-lactams, 65 (58%) aminoglycosides, and two (1.5%) fluoroquinolones. For β-lactams, 83% of the patients achieved a C_min_/MIC > 1 and 70% had a C_min_/MIC > 4. In the case of high creatinine clearance (CrCL > 100 mL/min/1.73 m^2^), 70.4% of the patients achieved a C_min_/MIC ratio > 1 versus 91% otherwise (*p* = 0.041), and 52% achieved a C_min_/MIC ratio > 4 versus 81% (*p* = 0.018). For aminoglycosides, 94% of the patients had a C_max_/MIC ratio > 8. Neither β-lactams nor aminoglycosides PK/PD parameters were associated clinical outcomes, but our data suggest a correlation between β-lactams C_min_/MIC and microbiological success. Conclusion: In our ICU patients treated for GNB VAP, using recommended antibiotic dosage led in most cases to PK/PD targets attainment for aminoglycosides and β-lactams. High creatinine clearance should encourage clinicians to focus on PK/PD issues.

## 1. Introduction

Ventilator associated pneumonia (VAP) remains a major cause of morbidity and mortality in critically ill patients despite advances in antimicrobial therapy and supportive care [1]. Convincing rationale suggests that for β-lactams, the duration of time that the antibiotic concentration remains above the MIC (T > MIC) during a dosing interval is the most relevant pharmacokinetic/pharmacodynamic (PK/PD) parameter predicting outcome, with classical values in intensive care unit (ICU) patients comprised between T > MIC = 100% and T > 6xMIC = 100% [2,3]. There are also strong data suggesting that aminoglycosides activity is optimal when maximum concentrations (C_max_) in serum reaches 8-10 fold above the MIC [4,5]. Concerning fluoroquinolones, both C_max_/MIC and area under the curve (AUC)/MIC ratios are related to the efficacy, with optimal values around 8–12 and 125–250 respectively [3,6]. Nevertheless, antibiotic therapy of VAP in ICU patients is challenging because of the wide pharmacokinetic variability combined to potentially high antibiotics MICs for Gram-negative bacilli (GNB) [7,8]. Previously published data pointed out that these PK/PD targets were not reached for ICU patients, highlighting the risk of “PD failure” [9,10,11]. However, to our knowledge, most studies defined the target concentrations of antibiotics, as well as the probability to attain the PK/PD targets, using the clinical breakpoints instead of measuring the actual MIC. Such an approach may lead to underestimating the probability of PK/PD target attainment. To our knowledge, few studies have reported the antibacterial effect of antibiotics based on PK/PD ratio using measured MICs instead of susceptibility breakpoints [12,13,14,15,16].

The objective of the present study was to assess the PK/PD targets attainment of antibiotics at their commonly prescribed doses, considering the measured MICs, in critically ill patients empirically treated for documented GNB VAP and to investigate the relationship between PK/PD endpoints attainment and outcome.

## 2. Results

### 2.1. Patient Characteristics

**Figure 1:**
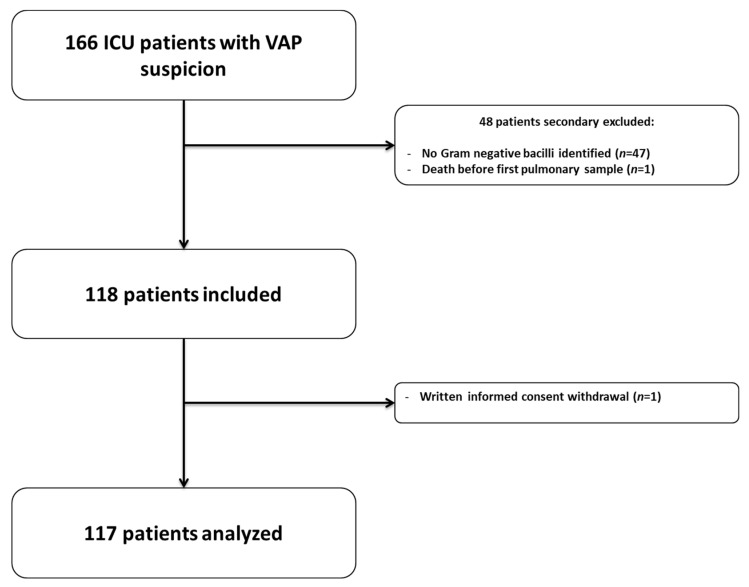
Flowchart.

One hundred and sixty-six patients with suspected VAP were enrolled; 49 were secondarily excluded and 117 were finally included (Figure 1). Patients’ characteristics are presented in Table 1. Median {IQR} age was 62 years {53–72}, and most patients were male (*n* = 87, 74%). At inclusion, median {IQR} SAPS2 and SOFA scores were 47 {38–61} and 8 {6–10}, respectively. Eighty-six patients (73%) had received antibiotics within the month preceding inclusion.

### 2.2. Microbiological Findings

Microbiological assessment was performed with broncho-alveolar lavage (BAL) for 17 patients (14.5%), plugged telescopic catheter (PTC) for 41 (35%), and tracheal aspirate (TA) for 59 (50.5%). Overall, 167 GNB were isolated from respiratory samples collected before treatment, including *Pseudomonas aeruginosa* (*n* = 48, 40%), *Enterobacter* spp. (*n* = 29, 23%) and *Escherichia coli* (*n* = 24, 20%) (Table 2). VAP was polymicrobial in 43 patients (37%). Median MICs of each antibiotic for all GNB species identified above cut-off values for respiratory samples and treated with the antibiotic of concern are presented in Table 3.

### 2.3. Antibiotic Therapy

When considering in vitro susceptibility, empirical antibiotic therapy was appropriate in 108 patients (96%). 

A β-lactam was used in 117 patients (100%), as monotherapy in 45 patients (38.5%), or in combination with an aminoglycoside in 68 patients (58%) or a quinolone in two patients (1.5%). Among β-lactams, piperacillin/tazobactam and cefepime were the most used drugs (45.5% and 24% of the patients, respectively) and continuous infusion was used for seven patients. Antibiotics’ dosing regimens for the 24 first hours of treatment are specified in Table 4. Total antibiotic treatment duration for VAP was eight days {6–9.5}. Antibiotic concentrations in plasma are presented in Supplementary Table S1.

### 2.4. Pharmacological Parameters

Finally, C_min_ of β-lactams were available in 102 patients among those definitely included (measured MICs were concomitantly available for 77 of them) and C_max_ of aminoglycosides or quinolones in 58 patients (measured MICs were concomitantly available for 48 of them). The PK/PD parameters of antibiotics are reported in Table 5.

**Figure 2:**
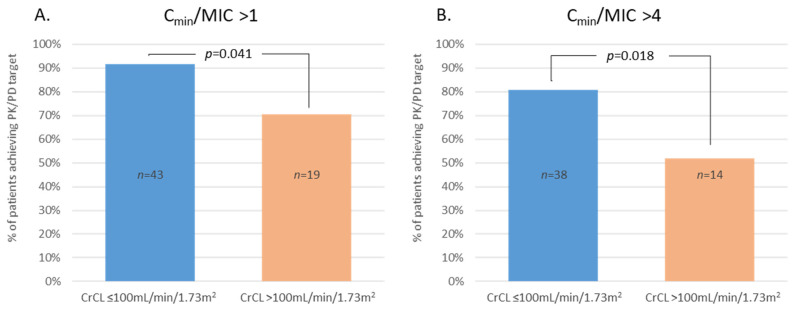
Proportion of patients achieving PK/PD targets for β-lactams, i.e., C_min_/MIC > 1 (**A**) or C_min_/MIC > 4 (**B**), according to CrCL.

For β-lactams, the median C_min_/MIC ratio at D1 was 12.6 {2.5–47.2}. Further, 64 patients (83%) achieved a C_min_/MIC ratio > 1 and 54 (70%) a C_min_/MIC ratio > 4 at D1. C_min_/MIC ratio was significantly lower in patient with high CrCL (>100 mL/min/1.73 m^2^): 4.8 {0.9–23.1} versus 14.8 {6–103.8}, *p* = 0.012. Among these patients, 19 (70.4%) achieved a C_min_/MIC ratio > 1 versus 43 patients (91%) without high CrCL (*p* = 0.041), and 14 (52%) achieved a C_min_/MIC ratio > 4 versus 38 patients (81%) without high CrCL (*p* = 0.018) (Figure 2).

For carbapenems, the median C_min_/MIC ratio at D1 was 5.4 {0.2–25}. Eight patients (61.5%) achieved a C_min_/MIC ratio > 1 and 7 (54%) a C_min_/MIC ratio > 4 at D1. For non-carbapenems β-lactams, the median C_min_/MIC ratio at D1 was 14 {3.2–75.6}. Further, 56 patients (87.5%) achieved a C_min_/MIC ratio > 1 and 47 (73%) a C_min_/MIC ratio > 4 at D1. There was no statistically significant difference between carbapenems and non-carbapenems β-lactams.

There was no difference in PK/PD target attainment between patients with monomicrobial VAP and those with polymicrobial VAP (data not shown).

The C_max_/MIC ratio for aminoglycosides, calculated for each infectious episode on D1, was above 8 in 44 VAP episodes (94%) and above 10 in 41 (87%) (Table 2). The median C_max_/MIC ratio was 32.5 {19.3–45.5}.

### 2.5. β-Lactams C_min_ Covariables

Age, SAPS2, CrCL, and presence of a high CrCL (>100 mL/min/1.73 m^2^) were significantly associated with the C_min_ in univariate analysis (Supplementary Table S2).

### 2.6. Microbiological Outcome

Microbiological success on day 3 occurred in 48 patients (62%). Table 6 presents the distribution of main PK/PD parameters according to the microbiological outcome. For β-lactams, 26 patients (93%) had a C_min_/MIC ratio > 1 in the microbiological success group compared to 19 (73%) in the microbiological failure group (*p* = 0.11). C_min_/MIC ratio was higher in the microbiological success group, 25.7 {6.9–119} versus 6.2 {1.1–46} (*p* = 0.041) (Table 6).

### 2.7. Clinical Outcome

After VAP episode, 35 patients (32%) needed a new antibiotic treatment until day 28, among whom seven experienced VAP recurrence. The median length of stay in intensive care unit and in hospital were 26 days {16–36} and 47 days {31–68}, respectively. Median mechanical ventilation duration was 18 days {12–24}. Overall mortality was 14.5% at D8 and 35% at D28.

At day 8, median SOFA and mCPIS scores were 5 {3–7} and 4 {3–5}, respectively. Clinical cure of VAP was obtained in 76% of the 70 patients with pharmacodynamic targets achieved for all antibiotics administered, 83% of the seven patients with only one pharmacodynamic parameter achieved (in case of combination therapy), and 71.4% of the eight patients with none of the pharmacodynamic parameters achieved (*p* = 0.88).

Patients alive at day 8 were significantly younger (61 {51.7–67} versus 68 {59–80}, *p* = 0.043), had a lower SOFA score at D1 (7 {5–9} versus 11 {9–13}, *p* < 0.001), and had higher CrCL (97 mL/min/1.73 m^2^ {65–115} versus 38 mL/min/1.73 m^2^ {33.5–88}, *p* < 0.001) than patients who had died. PK/PD parameters of β-lactams or aminoglycosides were not different between patients alive or dead at D8 (Table 5).

None of the other secondary outcomes (cure of VAP, poor clinical outcome or 28-day mortality) was significantly associated with the PK/PD of β-lactams or aminoglycosides (Supplementary Tables S3–S5), neither with the composite variable of PD target attainment taking into account combination therapy (data not shown).

## 3. Discussion

In this prospective study performed in critically ill patients with documented GNB VAP and receiving at least a β-lactam as empirical therapy, the overall PK/PD target attainment for β-lactams (C_min_/MIC ratio > 1 or >4) was 83% and 70% respectively at day 1, measuring both drug exposure and MICs for the causative pathogens. Furthermore, by excluding carbapenems that may allow less restrictive PK/PD targets due to the significant post-antibiotic effect on GNB [17], β-lactams PK/PD target attainment (C_min_/MIC ratio > 1 or >4) was 87.5% and 73%, respectively.

These data are not in accordance with previous studies that showed a higher risk of pharmacodynamic failure. Roberts et al. reported that C_min_/MIC ratio > 1 was achieved in only 60.4% of 361 critically ill patients treated various β-lactams [10]. Similarly, Carlier et al. concluded that a 3-h extended infusion of piperacillin/tazobactam (4 g/0.5 g) q.i.d was insufficient in critically ill patients to achieve the same pharmacodynamic target (C_min_/MIC > 1) throughout the entire treatment course and therefore could endanger treatment efficacy [18]. Taccone et al. found an adequate PD (i.e., C_min_/MIC > 4) for ceftazidime, cefepime and piperacillin/tazobactam in 28%, 16%, and 44% of their patients, respectively [11]. Patients’ characteristics in our study and those reported above seem similar especially in terms of severity (estimated by the SOFA score) and creatinine clearance, but those studies were only based on PD ratio calculated with clinical breakpoints.

In contrast, two recent studies using measured MICs instead of clinical breakpoints showed results very close to ours: Al-Shaer et al. showed in a retrospective monocentric study including 206 patients receiving β-lactams that C_min_/MIC ratio was above 1 in 89% of cases and above 4 in 72% [19]. Moreover, the TARGET trial [20] randomized 254 ICU patients receiving piperacillin/tazobactam in either a daily TDM-guided dose adjustment group or in a fixed dose group and found that, overall, approximately 27% of the patients failed to reach the PK/PD target at day 1, before adjustment. However, GNB were categorized in three groups according to MICs, and the highest MIC of each group was considered for target calculation (i.e., C_min_/MIC > 4). This may have led to an overestimation of PD failure. In our study, PD calculations took into account some resistant strains with high MICs (in respect of inclusion criteria) which may also have lowered the target attainment value. In particular, we observed lower C_min_/MIC and percentage of targets attainment in the carbapenems subgroup, likely due to resistant strains (MICs = 32 mg/L, Table 3) kept in this analysis in the case of polymicrobial VAP.

β-lactams pharmacodynamic failure, even when setting high targets (C_min_/MIC > 4), may thus be less common than reported in the literature. This points out the need for a better identification of patients with a high risk of pharmacodynamic failure.

Recent studies found a relatively high incidence, approximately 20%, of augmented renal clearance (ARC, commonly defined as a CrCL ≥ 130 mL/min/1.73 m^2^) in critically ill patients [21,22], which makes it a major concern. While different thresholds for ARC have been used in the literature, i.e., 120, 130, or 150 mL/min [23,24,25], Carrié et al. demonstrated in 79 septic critically ill patients that the best cut-off to predict underdosing (C_min_ < 4xMIC) was 170 mL/min [26]. It therefore appears difficult to set a single threshold, as there is a strong correlation, sometimes linear, between CrCL and clearance of primarily renally eliminated antibiotics, such as most β-lactams [27,28,29]: increasing the CrCL reduces the C_min_ and reduces subsequently the probability of target attainment (*f*T > MIC), particularly with more resistant organisms. Thus, some studies have demonstrated the need for dose escalation of ceftazidime [30], cefepime [31], meropenem [32], or piperacillin [29,33] as CrCL exceeded 90 or 100 mL/min, suggesting attention be paid to that lower range of CrCL. Nevertheless, authors did not only use measured MICs for PK/PD targets calculations, but also clinical breakpoints. In our study, CrCL was a covariate of β-lactams’ C_min_, as expected, and patients with high CrCL (>100 mL/min/1.73 m^2^) had significantly lower C_min_/MIC ratio. In addition, the proportion of patient achieving PK/PD targets, either C_min_/MIC > 1 or C_min_/MIC > 4, was significantly lower in case of high CrCL, as shown in Figure 2. Our study confirms previous results when using observed MICs, supposed to be lower than breakpoints, and should encourage clinicians to focus on this issue for lower-than-expected CrCL.

Our study suggests the importance of β-lactams PK/PD on the microbiological outcome. A significant higher mean C_min_/MIC ratio and a trend toward a higher target (C_min_/MIC > 1) attainment rate were indeed found in the microbiological success group. Conversely, no association was found between C_min_/MIC ratio and clinical outcome on D8 or D28. Montravers et al. showed that the microbiological outcome in VAP was predictive of the clinical outcome [34], but clinical outcomes in critically ill patients are linked to many factors, not only the appropriateness and adequacy of antimicrobial therapy, whereas microbiological outcomes are more closely dependent on the activity of the antimicrobial agent. Our negative result concerning clinical outcome has therefore to be seen in the light of the various confounding variables that may explain mortality in the ICU. For example, higher β-lactams C_min_/MIC ratios were found in our patients who died at D8 (non-significant difference, Table 5) but this could be related to lower CrCL, acute kidney injury being itself an independent risk factor for mortality [35], which was here also closely linked to much higher SOFA scores at D1. Similarly, since old preclinical studies [36,37,38], many recent trials assessing continuous infusion of β-lactams in ICU patients have failed to confirm the potential impact of antibiotics’ pharmacodynamics on clinical outcome [39,40,41]. Furthermore, optimal pharmacodynamic index ensuring clinical effectiveness is still debated as scientific evidence is variable, from C_min_/MIC > 1 to C_min_/MIC > 4–5 [10,42,43]. These studies, as our own, focused on β-lactams plasma concentrations whereas authors have suggested that standard dosing regimens could be insufficient to achieve optimal antibiotic concentrations in epithelial lining fluid [44], and this tissue diffusion concern could explain heterogeneous results. The combination with another antibiotic, most often an aminoglycoside in our trial, could also explain the lack of association, mainly because 94% of our patients reached a C_max_/MIC > 8, which is considered as an optimal target for aminoglycosides. Last, therapeutic drug monitoring (TDM) of β-lactams could be performed for each patient according to physician’s advice, so that individual PK/PD value at D1 may not reflect the C_min_/MIC obtained after dose adjustment.

The present study has several limitations. First, these analyses were performed using the total plasma concentration of β-lactams, without correction for protein binding. However, such a correction would have been of limited impact as the unbound fractions of the β-lactams used in the present study are usually >80% in healthy volunteers, and are likely higher in ICU patients due to hypoalbuminemia: 102.5% for ceftazidime, 98.4% for meropenem and 95.7% for piperacillin with interpatient variability of <6% [45]. Second, we did not measure antibiotic concentrations in the epithelial lining fluid. Nevertheless, for bedside TDM, measuring drug concentration at the site of infection could be less practical in critically ill patients. Third, the small sample size did not allow us to draw definite conclusions about how β-lactams pharmacodynamics impact either microbiological or clinical outcomes. Moreover, we had to deal with missing data, leading to a small cohort of patients with both MIC and C_min_ available, which decreased the statistical power. However, our results are quite similar to recent studies assessing PD targets achievement [19,20], and suggest that when a high β-lactams C_min_/MIC ratio is reached, optimal bacterial activity is achieved. Fourth, respiratory samples were not obtained with same method in all patients. Although European guidelines suggest obtaining distal quantitative samples (PTC or BAL in our study) for microbiological diagnosis of VAP in order to reduce antibiotic exposure [46], all methods are currently accepted [1,47]. Sampling techniques have similar accuracy in the diagnosis of VAP [47] and do not affect any clinical outcome [48]. Fifth, as our trial started and stopped before new drugs’ approval in France [49] and although there was no restriction in the antibiotic choice, our study focused mainly on piperacillin/tazobactam, cefepime, and carbapenems and therefore does not allow us to draw any formal conclusions on these new antibiotics. Nevertheless, PK/PD principles of β-lactams remain the same, especially in case of elevated CrCL [50,51]. Finally, we could not assess the impact of continuous infusion of β-lactams, since most patients received intermittent or extended infusions. However, although there is a theoretical benefit of continuous infusion to achieve higher plasma antibiotic concentrations [52], many recent trials have failed to confirm a potential impact of continuous infusion on clinical outcome [39,40,41].

## 4. Materials and Methods

### 4.1. Patients’ Selection

A multicentric prospective open-label trial was conducted in 11 French ICUs.

The ANSM (French National Agency for Medicines and Health Products Safety) registration number of the study was A120162-32/EudraCT 2012-000111-81. The protocol was approved by the Saint Germain En Laye Hospital Ethics Committee. The study was registered in CliniTrials.gov (NCT02127528). All patients or their relatives gave inform consent before enrollment.

ICU patients older than 18 years who had received mechanical ventilation for at least 48 h were eligible if they met all the following criteria: (i) clinical suspicion of VAP defined by a new or persistent infiltrate on chest radiography associated with at least 1 of the following: purulent tracheal secretions, temperature of 38.3 °C or higher, and leukocytes count higher than 10,000/mL; (ii) risk factor for acquired GNB with high antibiotic MICs, namely more than 5 days of mechanical ventilation, antibiotic treatment in the 15 days preceding infection, or known upper respiratory tract GNB colonization; (iii) a broncho-alveolar-lavage (BAL), a blinded plugged telescopic catheter (PTC), or tracheal aspirate (TA) has been performed as soon as VAP was clinically suspected. The cut-off values for GNB obtained from BAL, PTC and TA cultures were 10^4^ cfu/mL, 10^3^ cfu/mL and 10^6^ cfu/mL, respectively. Patients were secondarily excluded if no GNB reached these thresholds and the diagnosis of VAP was not retained by the clinician in charge, or all the GNB above these values were not susceptible to empirical antibiotic therapy, or renal replacement therapy (RRT) was performed between the two assays.

Patients were non included if they: (i) were pregnant, (ii) had a life expectancy ≤ 72 h following treatment initiation, or (iii) received first antibiotic administration > 24 h after diagnosis.

### 4.2. Drug Administration

Patients were empirically treated with a β-lactam and an aminoglycoside and/or a fluoroquinolone. Antibiotic choice, use of combination therapy, and dosing were left to the investigator’s decision but based on available recommendations at the time the trial was conducted. All doses were administered via an automatic high-precision infusion pump and the choice of the administration mode (short, extended, or continuous infusion) was left to the physician. Antimicrobial therapy was started within 24 h after microbiological sampling. Day 1 was defined as the first day of antibiotic therapy.

Physicians were encouraged to keep the empirical treatment unchanged for the first 48 h, then to de-escalate therapy towards antibiotics with a narrower spectrum. Treatment duration was left to the investigator’s choice, but 8-day therapy was suggested, with the exception of VAP caused by non-fermenting GNB, for which optimal treatment duration might be longer [53]. TDM of the prescribed ATB was allowed. Any adverse event related to treatment or its administration was recorded.

### 4.3. Data Collected

For each patient, the usual clinical and demographic data were collected at baseline, including age, gender, McCabe score [54], simplified acute physiology score (SAPS2) (admission and baseline) [55], sepsis-related organ failure assessment (SOFA) [56], and modified clinical pulmonary index score (mCPIS) [57]. At day 1, weight, fluid intake, vasopressor support, mechanical ventilation parameters, use of renal replacement therapy (RRT), and high flow oxygen or extracorporeal membrane oxygenation (ECMO) were noted, if applicable.

SOFA, vital status, and mechanical ventilation requirement were recorded at days 3, 5, and 8. Recurrence between end of treatment and day 28 were recorded. Duration of mechanical ventilation during ICU stay and vital status at day 28 were also recorded.

### 4.4. Assessment of Renal Function

The CKD-EPI equation was used to estimate creatinine clearance (CrCL) at day 1 [58]. High creatinine clearance was defined by a CrCL >100 mL/min/1.73 m^2^.

### 4.5. Blood Sampling and Analytical Method

Two blood samples were obtained for concentration measurement of each administered antibiotic: 0.5 h after the end of one of the infusions received during the first 24 first hours of treatment (C_max_) and just before the following infusion (through concentration, C_min_). Blood samples were immediately centrifuged within 30 min of collection, and the plasma was frozen at −80 °C until analysis in a centralized laboratory. For carbapenems, plasma was stabilized with the same volume of 1.0 M MOPS buffer, pH 7.0 (1:1) before being frozen at −80 °C. β-lactams concentrations in plasma were determined by a high-performance liquid chromatography with tandem mass spectrometry method. The limit of quantitation was 0.5 mg/L for all molecules.

Aminoglycoside’s concentrations in plasma were measured by fluorescence polarization immunoassay on the TDX analyzer (Abbott GmbH, Wiesbaden, Germany). The limit of quantitation was 0.5 mg/L.

### 4.6. Microbiological Analysis

A respiratory sample was performed as soon as VAP was clinically suspected, and before initiation of antimicrobial therapy and enrollment. Direct examination and quantitative culture on appropriate media were performed. Bacterial colonies were counted after 18–24 h of incubation. A second respiratory sample was performed after 48 h of treatment, at day 3, using the same methods. API systems (bioMérieux, Marcy-l’étoile, France) or MALDI-TOF Mass Spectrometry were used for bacterial identification and the disk diffusion method was used for antibiotic susceptibility testing. Antibiotics’ MICs were determined using Etest strips (bioMérieux, Marcy-l’étoile, France), according to the manufacturer’s instructions, for GNB isolated above defined thresholds (BAL ≥ 10^4^ cfu/mL, PTC ≥ 10^3^ cfu/mL or TA ≥ 10^6^ cfu/mL) from first samples (before administration of antibiotics). Antibiotic MICs or diameters were interpreted according to the recommendations of the European Committee on Antimicrobial Susceptibility Testing (EUCAST).

### 4.7. Pharmacodynamic Analysis

Several pharmacodynamic parameters were determined for each patient and antimicrobial. For β-lactams, we analyzed the following parameters: C_min_/MIC, number of patients achieving a T > MIC of 100% (C_min_/MIC ratio > 1) and number of patients achieving a T > 4xMIC of 100% (C_min_/MIC ratio > 4). For aminoglycosides and fluroquinolones pharmacodynamic analysis, we studied the C_max_/MIC ratio, and the number of patients achieving a C_max_/MIC > 8 or 10 for aminoglycosides and >10 for quinolones.

To take into account the role of combination therapy in the clinical course (β-lactam + aminoglycoside or β-lactam + fluoroquinolone), a qualitative variable based on the simultaneous attainment of T > MIC = 100% for β-lactams and C_max_/MIC > 8 for aminoglycosides or >10 for fluoroquinolones was created. Patients were included in 3 groups according to the achievement of these targets (none, 1, or 2 of the pharmacodynamic targets achieved).

In the case of a polymicrobial infection, patient-specific pharmacodynamic parameters were computed considering the highest MIC among GNB recovered at inclusion.

### 4.8. Outcome Assessment

The primary endpoint of the study was the proportion of patients achieving the pharmacodynamic targets defined above, using both MICs and concentrations measurements at the first day of treatment.

Several secondary endpoints were also assessed: microbiological success (defined as none of the GNB species identified at D1 grown above cut-off values at D3), 8- and 28-day mortality, cure of VAP defined as CPIS < 6 and alive at day 8, poor clinical outcome (defined as a composite criterion: SOFA score > 3 or death at day 8), and total duration of mechanical ventilation during ICU stay. We also evaluated the impact of PK/PD parameters on the microbiological and clinical outcomes.

### 4.9. Statistical Analysis

The statistical analysis was performed on the cohort of included subjects who have confirmed all inclusion and non-inclusion criteria (including secondary exclusion criteria). Results are presented as median {interquartile range} (minimum; maximum) for quantitative data and count (percentage) for categorical data.

The proportion of pharmacodynamic success was estimated with its 95% confidence interval. Analysis of secondary objectives included estimation (pointwise with 95% confidence interval) of the proportion of pharmacological success by detailing the 3 different pharmacodynamic targets of the primary endpoint and search for an association.

For each outcome, a comparison of patient’s characteristics and of PK/PD parameters was performed using student t-test for quantitative data and Chis-square test for qualitative data. Of note, due to their skewed distribution, PK/PD parameters, such as C_min_, C_max_, C_min_/MIC, and C_max_/MIC, were log-transformed before being compared.

A *p*-value < 0.05 was considered significant. All analysis were performed with R (version 4.0.4 (15 February 2021)) and SAS (version 9.3) softwares.

## 5. Conclusions

In conclusion, in a setting of documented GNB VAP receiving at least a β-lactam as empirical therapy, 83% of our patients achieved an appropriate pharmacodynamic target for β-lactams, defined as C_min_/MIC > 1, and patients with high CrCL >100 mL/min/1.73 m^2^ had a significantly lower proportion of target attainment, which should encourage clinicians to focus on this issue. No correlation was found between β-lactams C_min_/MIC ratio and clinical outcome, but our data suggest a correlation between C_min_/MIC and microbiological success.

These findings suggest that an exclusive focus on C_min_ without MIC measurement is probably not suitable to evaluate the pharmacodynamics efficacy of antibiotic therapy and leads to overstating the risk of PD failure. The routine assessment of β-lactam pharmacodynamic seems nevertheless interesting, as the overall probability of pharmacodynamic failure was still 17%, and up to 30–50% (depending on the target) in case of high CrCL.

## Figures and Tables

**Table 1:** Demographics and clinical characteristics of the 117 patients at baseline and day 1.

	*Value*
Age (years)	62 {53–72}
Female	30 (26)
Weight (kg)	75 {61–90}
Body Mass Index (kg/m^2^)	25.6 {22–29.5}
SAPS2 at admission	54 {43–64}
*Admission category* Medical	90 (77)
Surgical	27 (23)
*McCabe classification* 1	89 (81.5)
2	17 (15.5)
3	4 (3)
*Reason for admission*	
Respiratory failure	42 (36)
Hemodynamic failure	26 (22)
Sepsis	10 (8.5)
Abdominal disease/Hepatic failure	3 (3)
Acute renal failure/Metabolic disease	2 (2.5)
Neurologic failure	31 (26.5)
Other	2 (2)
*Clinical presentation at VAP diagnosis or D1*	
*Reason for mechanical ventilation*	
Cardiac arrest	18 (15)
Surgery	12 (10)
Septic shock	4 (3)
Hemorrhagic shock	1 (1)
Cardiogenic shock	4 (3.5)
Acute respiratory failure	43 (37)
Toxic or metabolic coma	4 (3.5)
Neurologic failure	24 (20)
Other	7 (6)
Time intubation—VAP (days)	9 {6–11}
Temperature (°C)	38.5 {37.8–39.1}
mCPIS	6 {5–7}
SAPS2	47 {38–61}
SOFA	8 {6–10}
Vasopressor	45 (38.5)
PaO_2_/FiO_2_ ratio (mmHg)	167 {108–240}
Leukocytes (G/L)	15 {11–19}
Serum creatinine at D1 (µmol/L)	68.5 {48–114}
Creatinine clearance at D1 (mL/min/1.73 m^2^)	94 {54–112}
Serum albumin at D1 (g/L)	19 {15–24}
Weight (kg)	78.5 {65–92}
RRT (D-2 to D0)	1 (1)
Fluid intake during D1 (mL)	2477 {1485–3450}

VAP, ventilator-associated pneumonia; SAPS2, simplified acute physiology score; SOFA, sepsis-related organ failure assessment; mCPIS, modified clinical pulmonary index score. Data are presented as median {IQR} or n (%).

**Table 2:** Microbiological findings in the first respiratory sample obtained (before treatment).

	*Value*
*Respiratory sample*	
Broncho Alveolar Lavage	17 (14.5)
Plugged Telescopic Catheter	41 (35)
Tracheal Aspirate	59 (50.5)
Positive blood culture	9 (8)
Polymicrobial VAP	43 (37)
All GNB identified (*n = 167*)	
*P. aeruginosa*	48 (40)
*Enterobacter* spp.	29 (23)
*E. coli*	24 (20)
*Klebsiella* spp.	19 (16)
*Haemophilus influenzae*	10 (8)
*Stenotrophomonas maltophila*	6 (5)
*Citrobacter* spp.	5 (4)
*Morganella* spp.	5 (4)
*Acinetobacter* spp.	4 (4)
*Serratia* spp.	4 (3)
*Proteus* spp.	4 (3)
Others	9 (8)

MIC, minimal inhibitory concentration; GNB, Gram-negative bacilli; VAP, ventilator-associated pneumonia. Data are presented as n (%).

**Table 3:** Median MICs of each antibiotic for all GNB species identified above cut-off values for respiratory samples and treated with the antibiotic of concern.

	*Value*	
	*n*	*MIC*
*β-lactams*		
*Piperacillin/tazobactam*	41	2 {0.85–4} (0.2;256)
*Cefepime*	22	0.1 {0.0395–1} (0.023;4)
*Ceftazidime*	9	1.5 {0.8–13} (0.1;256)
*Imipenem*	9	0.35 {0.2–2.62} (0.1;32)
*Meropenem*	8	0.2 {0.043–1.05} (0.023;32)
*Piperacillin*	*NA*	
*Cefotaxime*	*NA*	
*Aminoglycosides*		
*Amikacin*	52	2 {1.5–3} (0.4;256)
*Tobramycin*	1	0.9
*Quinolones*		
*Ciprofloxacin*	2	0.094 (0.032;6.4)

MIC, minimal inhibitory concentration. Data are presented as median {IQR} (min;max) or n (%).

**Table 4:** Empirical antibiotic therapy and dose regimens.

	*Value*		
	n (%)	*Daily Dose Regimen*	
*Number of antibiotics per patient*			
1	45 (38.5)		
2	70 (60)		
3	2 (1.5)		
Antibiotics and dose regimen			
*β-lactams*	117 (100)	*(dose in g/day)*	*CI* *
*Piperacillin/tazobactam*	53 (45.5)	16/2 {12/1.5–16/2} (8/1;20/2.5)	
*Cefepime*	28 (24)	6 {6–6} (4;8)	
*Ceftazidime*	12 (10)	6 {6–6} (6;6)	6 {6-6} (4;8)
*Imipenem*	13 (11)	3 {3–3} (2;4)	
*Meropenem*	8 (7)	5 {3–6} (1;6)	3
*Piperacillin*	2 (2)	16 (16;16)	
*Cefotaxime*	1 (0.5)	8	
*Aminoglycosides*	68 (58)	*(dose in mg/kg/day)*	
*Amikacin*	67 (57)	26 {23–29} (16;35.5)	
*Tobramycin*	1 (0.5)	9.5	
*Quinolones*	2 (1.5)	*(dose in mg/day)*	
*Ciprofloxacin*	2 (1.5)	1200 (1200;1200)	

*: continuous infusion. Data are presented as median {IQR} (min;max) or n (%).

**Table 5:** Pharmacokinetic and pharmacodynamic parameters of the patients and comparison according to clinical outcome.

	*Overall Population*	*Alive at D8*	*Death at D8*	*p*
β-lactams	*n = 77*	*n = 68*	*n = 9*	
C_min_/MIC	12.6 {2.5–47.2} (0.001;637)	11.6 {1.9–60} (0.001;637)	20.8 {7.5–46} (3.2;116.5)	0.21
C_min_/MIC > 1	64 (83%)	55 (81)	9 (100)	0.34
C_min_/MIC > 4	54 (70%)	46 (68)	8 (89)	0.27
*Aminoglycosides*	*n = 47*	*n = 40*	*n = 7*	
C_max_/MIC	32.5 {19.3–45.5} (0.17;165)	32.4 {18.2–44.5} (0.17;165)	33.4 {21.4–44} (0.26;126.9)	0.63
C_max_/MIC > 8	44 (94)	38 (95)	6 (86)	0.39
C_max_/MIC > 10	41 (87)	35 (87.5)	6 (86)	1.00

Data are presented as median {IQR} (min;max) or n (%).

**Table 6:** Comparison of pharmacokinetic/pharmacodynamic parameters according to microbiological outcome on day 3.

	*Microbiological Failure*	*Microbiological Success*	*p*
β-lactams	*n = 26*	*n = 28*	
C_min_/MIC	6.2 {1.1–46} (0.001;513)	25.7 {6.9–119} (0.4;637)	0.041
C_min_/MIC > 1	19 (73)	26 (93)	0.11
C_min_/MIC > 4	17 (65)	23 (82)	0.27
*Aminoglycosides*	*n = 17*	*n = 20*	
C_max_/MIC	24.4 {19.6–37} (0.26;61.8)	35 {21–51} (0.18;165)	0.91
C_max_/MIC > 8	16 (94)	18 (90)	1.00
C_max_/MIC > 10	16 (94)	16 (80)	0.44

Data are presented as median {IQR} (min;max) or n (%).

## Data Availability

Data available on request.

## Supplementary Materials

The following supporting information can be downloaded at: https://www.mdpi.com/article/10.3390/antibiotics11111664/s1, Table S1: Antibiotics’ plasma concentration measurements. Data are presented as median [IQR] (min; max); Table S2: Covariables of β-lactams’ log(C_min_) Univariate analysis title; Table S3: Pharmacokinetic and pharmacodynamic parameters of the patients and comparison according to clinical outcome (cure of VAP); Table S4: Pharmacokinetic and pharmacodynamic parameters of the patients and comparison according to clinical outcome; Table S5: Pharmacokinetic and pharmacodynamic parameters of the patients and comparison according to clinical outcome (28-day mortality).
