# Supplementary material for: Empirical Antibiotic Therapy for Gram-Negative Bacilli Ventilator-Associated Pneumonia: Observational Study and Pharmacodynamic Assessment
Source: Antibiotics (Basel). 2022 Nov 19;11(11):1664. doi: 10.3390/antibiotics11111664 (PMC9686941; doi:10.3390/antibiotics11111664)
Supplement: Supplementary file 1 [file antibiotics-11-01664-s001.zip › antibiotics-1997923-supplementary.pdf]

Supplementary data

Table S1. Antibiotics' plasma concentration measurements. Data are presented as median {IQR} (min; max)

	Value	
	n	C(mg/L)
β-lactams		C_{min}
Piperacillin (tazobactam)	41	12.3 {4.8–42.2} (1.43;233)
Cefepime	22	12.6 {7–23.8} (3.3;57.4)
Ceftazidime	9	41.6 {26.6–52.7} (2.9;60)
Imipenem	9	2.1 {1.3–2.2} (0.5;2.7)
Meropenem	8	8 {4.6–11.3} (0.03;25.4)
Aminoglycosides		C_{max}
Amikacin	52	71.2 {61.7–95.3} (40;316)
Tobramycin	1	21.4
Quinolones		C_{max}
Ciprofloxacin	2	5.1 (5.1;5.1)

Table S2. Covariables of β-lactams' log(C_{min}) Univariate analysis

Variable	N	*n*	*Beta (95%CI)*	*p*
Age	148		0.03(0.02; 0.05)	<0.0001
Weight	145		0 (-0.01; 0.01)	0.93
Oedema score	94		0 (-0.06; 0.05)	0.89
Fluid intake during D1 (*100mL)	139		0 (-0.02; 0.01)	0.85
Creatinine clearance at D1 (mL/min)	145		-0.02 (-0.03 ;-0.02)	<0.0001
High renal clearance				
No	91		0	
Yes (>100mL/min/1.73m^2)	54		-1.4 (-1.8;-0.99)	<0.0001
PaO_2/FiO_2 ratio	146		0.01 (-0.02; 0.03)	0.58
SAPS2 (admission)	146		0.02 (0.01; 0.03)	0.004
SOFA score (day 1)	146		0.06 (-0.01; 0.12)	0.086
Vasopressor				
Yes		63	0.19 (-0.25; 0.64)	0.39
No		85	0	
RRT				
Yes		2	-0.13 (-2.06; 1.81)	0.90
No		135	0	
Continous infusion				
Yes		10	0	
No		138	-0.73 (-1.61; 0.14)	0.10

Table S3. Pharmacokinetic and pharmacodynamic parameters of the patients and comparison according to clinical outcome (clinical cure of VAP). Data are presented as median {IQR} (min;max) or n (%).

	No cure of VAP	*Cure of VAP*	*p*
β-lactams	*n=16*	*n=52*	
C_{min}/MIC	15.4{4.4;61} (0.07;183)	11.6 {1.9;49.5} (0.18;637)	0.89
C_{min}/MIC >1	14 (88)	43 (83)	0.94
C_{min}/MIC >4	12 (75)	35 (67)	0.78
Aminoglycosides	*n=9*	*n=34*	
C_{max}/MIC	28.6 {17.0;42.5} (0.17;165)	34.3 {19.6;51.7} (0.26;126.9)	0.85
C_{max}/MIC >8	8 (89)	32 (94)	1.00
C_{max}/MIC >10	8 (89)	30 (88)	1.00

Table S4. Pharmacokinetic and pharmacodynamic parameters of the patients and comparison according to clinical outcome. Data are presented as median {IQR} (min;max) or n (%).

	Poor clinical outcome	*Good clinical outcome*	*p*
β-lactams	*n=30*	*n=14*	
C_{min}/MIC	19.6 {4.2;67.9} (0.07;637)	3.1 {1.2;14.1} (0.18;431.9)	0.07
C_{min}/MIC >1	26 (87)	10 (71)	0.42
C_{min}/MIC >4	23 (77)	6 (43)	0.06
Aminoglycosides	*N=16*	*n=11*	
C_{max}/MIC	34.6 {21.6;42.7} (8.2;158)	32.3 {11;49.2} (0.17;165)	0.22
C_{max}/MIC >8	16 (100)	9 (81)	0.31
C_{max}/MIC >10	15 (94)	8 (73)	0.34

Table S5. Pharmacokinetic and pharmacodynamic parameters of the patients and comparison according to clinical outcome (28-day mortality). Data are presented as median {IQR} (min;max) or n (%).

	Overall population	*Alive at D28*	*Death at D28*	*p*
β-lactams	*n=77*	*n=49*	*n=28*	
C_{min}/MIC	12.6 {2.5–47.2} (0.001;637)	12.6 {2.5 – 47} (0.10;534)	12.7 {2.6 – 62.3} (0.001;637)	0.68
C_{min}/MIC >1	64 (83%)	43 (88)	21 (75)	0.26
C_{min}/MIC >4	54 (70%)	34 (69)	20 (71)	1.00
Aminoglycosides	*n=47*	*n=30*	*n=17*	

C_{max}/MIC	32.5 {19.3–45.5} (0.17;165)	28.6 {17.0;42.5} (0.17;165)	34.3 {19.6;51.7} (0.26;126.9)	0.63
C_{max}/MIC >8	44 (94)	28 (93)	16 (94)	1.00
C_{max}/MIC >10	41 (87)	25 (83)	16 (94)	0.78
